# Infarcts of a Cardioembolic Source Mimicking Lacunar Infarcts: Case Series With Clinical and Radiological Correlation

**DOI:** 10.7759/cureus.43665

**Published:** 2023-08-17

**Authors:** Ahmed Harazeen, Muhammad Z Memon, Heitor Frade, Arun Chhabra, Umar Chaudhry, Hashem Shaltoni

**Affiliations:** 1 Neurology, University of Texas Medical Branch, Galveston, USA; 2 Radiology, University of Texas Medical Branch, Galveston, USA

**Keywords:** heart catheterization complications, clinical correlation, radiological correlation, embolic source, lacunar infarcts

## Abstract

Lacunar strokes are the hallmark of cerebral small vessel disease. There are several well-established mechanisms for the pathogenesis of lacunar stroke, but the cardioembolic mechanism is not well-established. Three cases of acute ischemic stroke following elective cardiac and cerebral catheterization are reported. These cases had typical lacunar-looking infarcts on neuroimaging despite strong evidence of an embolic source with temporal correlation. Awareness of such findings and pathogenesis may help investigational workup and management of these patients.

## Introduction

Lacunar strokes account for up to a quarter of all ischemic strokes and measure less than 20 mm in diameter. They are usually caused by the occlusion of a perforator of an intracranial artery [1]. There are several well-established mechanisms for the pathogenesis of lacunar stroke, including lipohyalinosis, atherosclerotic disease, and branch atheromatous disease [1-3]. Another pathogenic mechanism that is not well-known is cardiac embolism [4]. Lacunar strokes typically have infarcts involving deep grey matter nuclei, periventricular white matter, pons, or cerebellar hemispheres. Some studies have suggested that cardioembolic mechanisms are unlikely to cause lacunar strokes [5,6]. Three cases of post-procedural stroke are discussed, in which the correlating imaging findings are consistent with lacunar infarction but had a preceding embolic source, thereby suggesting a cardioembolic source as another mechanism of lacunar infarcts.

## Case presentation

Case 1

**Figure 1:**
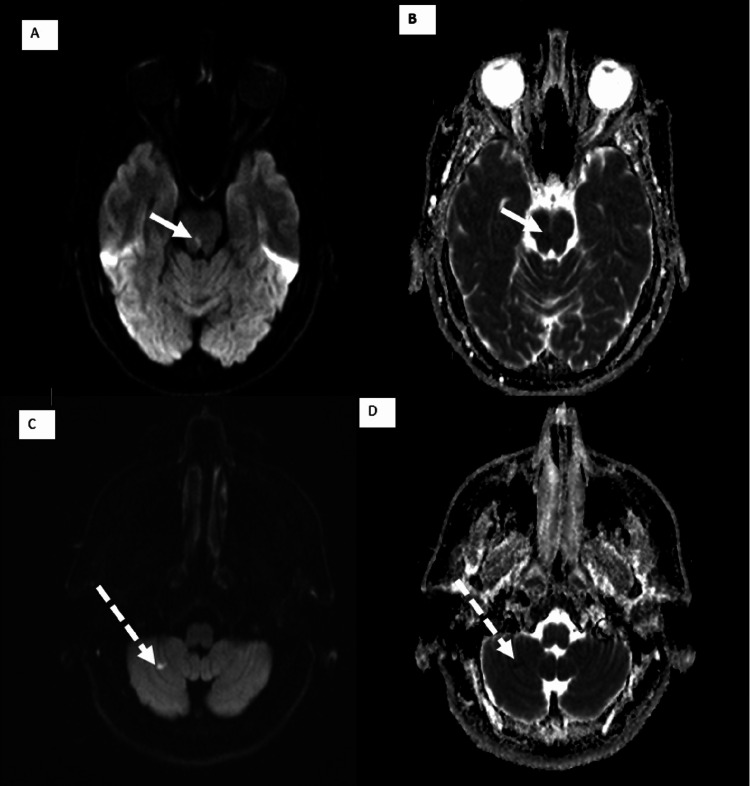
Axial MRI images (A,B) DWI and ADC showing an acute small right posterior pontine infarct (white arrows) (C,D) DWI sequence and ADC showing an acute small infarct in the right cerebellar region (dashed arrows) DWI, diffusion-weighted image; ADC, apparent diffusion coefficient

A 58-year-old Caucasian woman with multiple vascular risk factors, including hypertension and hyperlipidemia, presented to the hospital for an elective diagnostic cardiac catheterization after she was found to have unstable angina a few days earlier. After the diagnostic part, angioplasty of the left anterior descending artery was performed with full-dose heparin anticoagulation. She began endorsing diplopia immediately post-procedure. A neurological exam showed impaired adduction of the right eye with preserved convergence, consistent with right medial rectus palsy. Her National Institutes of Health Stroke Scale (NIHSS) score was 1. CT head without contrast showed an Alberta stroke program early CT score (ASPECTS) of 10. CT angiograms of the head and neck were notable for mild atherosclerotic disease in bilateral cavernous segments of internal carotid arteries without flow-limiting stenosis or large vessel occlusion. She was not deemed a candidate for systemic thrombolysis due to heparin use during the procedure with an aPTT of 62 at the time of evaluation and mild deficit. MRI brain without contrast showed acute small infarcts in the right posterior midbrain suggestive of lacunar small vessel stroke and right cerebellum infarct (confirms catheter-related embolic etiology) (Figure 1).

A 2-D echocardiogram was unremarkable with no interatrial shunt. Her diplopia improved over the next few days, and she was discharged on antiplatelet therapy. At the three-month follow-up, her NIHSS score was 0 with a modified Rankin score of 1.

Case 2

**Figure 2:**
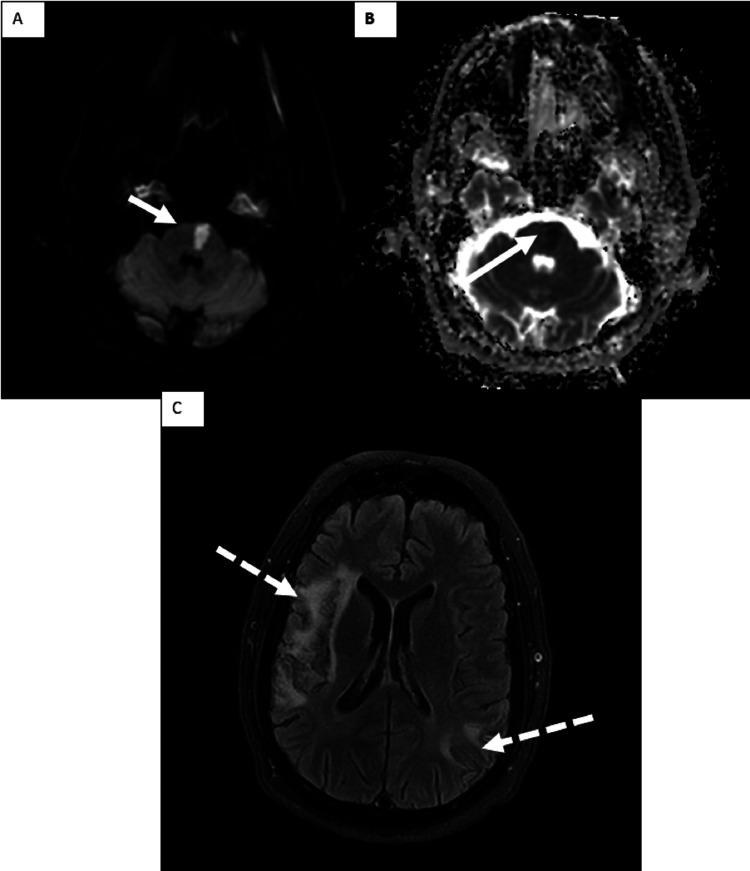
Axial MRI images (A,B) DWI and ADC showing an acute left pontine infarct (white arrow) (C) FLAIR sequence showing bilateral chronic infarcts in the right middle cerebral artery territory (right frontal operculum and right temporal lobe with a peripheral watershed infarct in the left parietal-occipital junction) (two dashed arrows) DWI, diffusion-weighted image; ADC, apparent diffusion coefficient

A 54-year-old Hispanic man with a history of hypertension, hyperlipidemia, and diabetes mellitus, with a baseline modified Rankin score of 2, was admitted to the neurology service after developing sudden onset left-sided weakness following an elective cerebral angiogram that was done to evaluate intracranial and extracranial vasculature given prior bilateral cortical strokes. He had no family history of stroke or other neurological conditions. The patient didn’t have any new neurological complaints immediately post-op and was moving all extremities and had a meal. A few hours later, he was found to have a right facial droop, right-sided weakness, and anarthria. His NIHSS score was 11. He was out of the IV t-PA time window. A CT brain showed bilateral chronic infarcts, and the CTA of the head and neck showed diffused intracranial and extracranial moderate atherosclerotic disease, which was not flow-limiting. There was no large vessel occlusion. CT perfusion did not reveal hypoperfusion. MRI brain showed a small acute infarct in the left hemi-pons and small chronic infarcts in the middle cerebral territory bilaterally (Figure 2). Transthoracic echocardiogram showed a large echogenic mass of 2.6 cm × 2.0 cm attached to the interatrial septum and dropping into the left ventricle during the diastolic opening of the anterior mitral leaflet, concerning atrial myxoma. A complete transesophageal echocardiogram was performed and confirmed the findings. The patient was initially started on a heparin drip, which was bridged to Coumadin. The patient did not have any meaningful improvement in his neurological exam and had a modified Rankin score of 5 at three months.

Case 3

**Figure 3:**
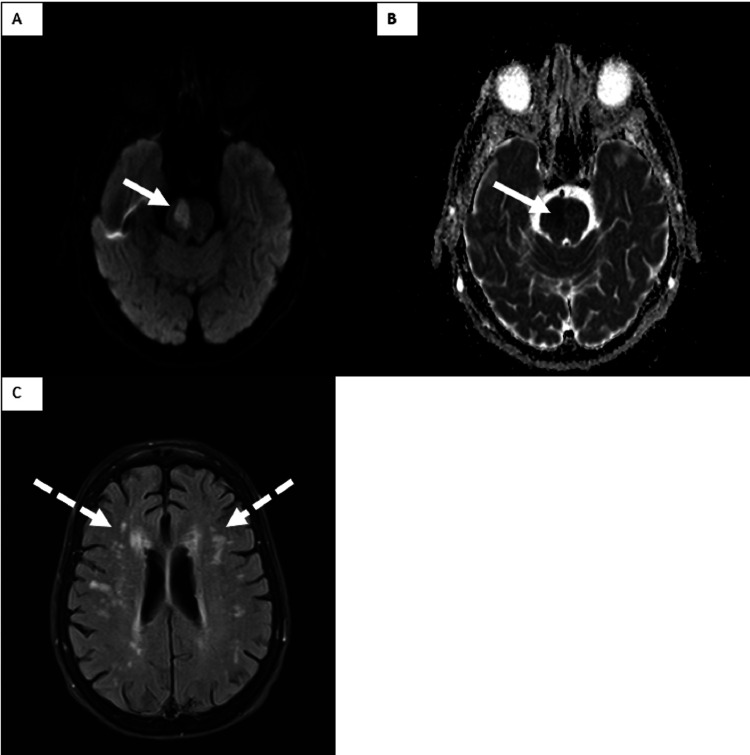
Axial MRI images (A,B) DWI and corresponding ADC showing an acute right hemi-pons lesion (solid arrow) (C) FLAIR sequence showing multiple T2/FLAIR hyperintensities in a supratentorial deep, subcortical, and periventricular white matter consistent with chronic microvascular ischemic changes (dashed arrows) DWI, diffusion-weighted image; ADC, apparent diffusion coefficient

A 76-year-old right-handed female with a history of hypertension, hyperlipidemia, type 2 diabetes mellitus, chronic kidney disease stage II, COPD, and current smoking was admitted to the hospital for elective left heart catheterization (LHC) to work up coronary artery disease. LHC was performed with right radial access and noted severe atherosclerotic aortic arch disease and three-vessel coronary artery disease. Systemic anticoagulation was not administered, and no intraprocedural complications were noted. The patient was then transferred to the recovery area, where she was observed to have an inability to raise her left arm about 20 minutes after the procedure. A code stroke was activated, and the patient was found to have left-sided weakness, ataxia, and dysarthria with an NIHSS score of 5 and blood pressure of 180/67 mmHg. A CT brain showed an ASPECTS score of 10. The CTA of the head and neck showed mild to moderate extracranial and intracranial atherosclerotic disease without flow-limiting stenosis or large vessel occlusion. The patient received intravenous t-PA. MRI brain showed a moderate area of diffusion restriction in the right hemi-pons. Additionally, there were supratentorial chronic microvascular ischemic changes without brainstem involvement. Transthoracic echocardiogram with a bubble study showed no interatrial shunt with impaired relaxation and an estimated EF of 60-65%. At the three-month follow-up, the patient’s NIHSS score improved to 3, and the modified Rankin score was 2 (Figure 3).

## Discussion

Lacunar strokes are largely due to the pathologic consequences of underlying cerebral small vessel disease [7-9]. Even though emboli from cardiac or other proximal sources can cause occlusion of deep perforating arteries resulting in lacunar infarction, most lacunar strokes are due to white matter disease [10,11]. A study of monkey brains showed that direct injection of emboli into the internal carotid artery resulted in infarcts involving mostly circumferential arteries, but in a small proportion of cases, it also resulted in deep perforating artery lacunar infarcts [12]. Park et al. [13], from their series of 231 patients with atrial fibrillation and lacunar infarcts on MRI, showed that acute small deep infarction was likely caused by intrinsic small vessel disease rather than being cardioembolic despite the presence of concomitant atrial fibrillation. Another argument against embolic etiology is that lacunar infarcts are less likely to occur in the setting of a patent foramen ovale [14]. Therefore, current evidence suggests that lacunar stroke is a very rare manifestation of cardioembolism. In comparison, stroke due to embolic etiology involves more than one vascular territory and is often multiple [15]. Periprocedural strokes involving cardiac catheterization or cerebral angiography are thought to be primarily related to artery-to-artery embolism [16-18]. Thrombus formation in the catheter or on its surface or dislocation of aortic atheroma during manipulation and passage of catheters within the aorta are the main sources of embolic material during these procedures [16,19]. Additionally, air embolism and scraping of aortic plaques during catheter navigation can lead to the embolization of debris to the brain, leading to ischemic stroke. Typical CT or MRI findings for periprocedural stroke would have cortical, subcortical, or posterior fossa infarcts greater than 15 mm in diameter involving multiple territories.

Three cases of cerebrovascular events after elective diagnostic cardiac and cerebral angiograms are reported. Patients developed symptoms that were recognized post-operatively, and an MRI brain showed findings of acute stroke located in typical lacunar areas involving white matter and brainstem supplied by small penetrating arteries. The proposed mechanism of ischemic stroke in the first case was embolic from artery-to-artery embolism to perforator branches involving vertebrobasilar circulation. In the second case, a paramedian pontine area infarct is found. The patient was found to have a large atrial myxoma extended to the left ventricle, a known high-risk cardioembolic source, and the patient was also found to have chronic strokes on the contralateral territory. In this case, two potential causes of stroke were secondary to the cardioembolic source from the atrial myxoma and periprocedural cerebral angiography. The third patient received t-PA with clinical improvement, but the MRI brain showed a right hemi-pons infarct. The proposed cause was embolic material from extensive aortic arch disease, which got dislodged during cardiac catheterization. Therefore, acute infarcts were observed on MRI images, which would typically represent small vessel stroke patterns but with a known history of embolic etiology. Despite well-defined clinical and temporal causative factors for cardioembolic in these series, these associations may be due to shared risk factors of small vessel disease, such as hypertension, dyslipidemia, diabetes mellitus, and cigarette smoking. It is important to recognize cardioembolic etiology in lacunar-appearing infarcts, as this was diagnostic and prognostic implications. In addition, their responses to treatment with an antithrombotic or anticoagulant drug may be distinct and may involve careful clinical decision-making.

## Conclusions

Neuroimaging findings of small vessel lacunar strokes typically involve periventricular deep white matter, deep grey matter nuclei, and brainstem. Thromboembolic strokes typically appear cortical and usually multiple within the same arterial territory or multiple territories. The three cases have shown embolic proven infarcts looking like lacunar infarcts on neuroimaging. Awareness of such findings on imaging may help during investigational stroke workup and subsequent treatment.
